# “Seconds save lives—clean your hands”: the 5 May 2021 World Health Organization *SAVE LIVES: Clean Your Hands* campaign

**DOI:** 10.1186/s13756-021-00926-7

**Published:** 2021-03-17

**Authors:** Benedetta Allegranzi, Ermira Tartari, Didier Pittet

**Affiliations:** 1 Infection Prevention and Control Technical and Clinical Hub, Department of Integrated Health Services, World Health Organization (WHO), Geneva, Switzerland; 2 Faculty of Health Sciences, University of Malta, Msida, Malta; 3 Infection Control Programme, University of Geneva Hospitals and Faculty of Medicine, Gabrielle-Perret-Gentil 4, 1205 Geneva, Switzerland

The year 2020 was unprecedented in many ways, one of which was the tremendous attention given to appropriate hand hygiene practices in the fight against SARS-CoV-2. Hand hygiene has finally gained global recognition from policy-makers, health managers, health care workers and the general public as a keystone in infection prevention. The World Health Organization (WHO) placed increased focus on hand hygiene in the context of the global COVID-19 pandemic, in addition to its longstanding efforts through both the global *SAVE LIVES: Clean Your Hands* campaign and the Water, Sanitation and Hygiene (WASH) Programme.

In the context of the pandemic WHO launched several initiatives, including the new WHO and UNICEF Hand Hygiene for All initiative (https://www.who.int/water_sanitation_health/sanitation-waste/sanitation/hand-hygiene-for-all/en/) to consistently improve hand hygiene practices as a whole-of-society approach to stop the spread of SARS-CoV-2 and sustain good practices beyond the pandemic. To achieve these goals, adequate infrastructures should be provided in health care and public settings, including for example schools and public transportations, and appropriate behaviour to clean hands when needed should be taken by all key players.

This year, WHO’s *SAVE LIVES: Clean Your Hands* campaign focuses on achieving appropriate hand hygiene action at the point of care. This has been at the core of WHO patient safety strategies during health care delivery for many years, but is now more critical than ever. Furthermore, 2021 has been designated the International Year of Health and Care Workers (https://www.who.int/campaigns/annual-theme/year-of-health-and-care-workers-2021): focusing on their protection is also paramount.

The first prerequisite for effective implementation of hand hygiene action at the point of care is “system change” meaning that the appropriate infrastructure and supplies should be available at the point of care so that health workers can clean their hands promptly when needed. This requires reliable and uninterrupted provision of good-quality alcohol-based handrub (ABHR), supplies of clean water, soap, single-use towels and an adequate number of functioning sinks. Although effective infection prevention and control (IPC) programmes in health care facilities should meet WHO minimum requirements [1], the 2020 global WASH report revealed that one in three facilities do not have adequate hand hygiene stations at the point of care [2]. A recent systematic review showed that hand hygiene compliance is only around 9% during care of critically ill patients in low-income countries [3]; such shocking data, in conjunction with the ongoing COVID-19 pandemic, highlight the urgent need for additional efforts to strengthen global compliance and champion best practices. Although vaccines are starting to be delivered, hand hygiene and appropriate use of personal protective equipment remain crucial for safe care of both COVID-19 and non-COVID-19 patients.

Effective hand hygiene not only reduces the burden of health care-associated infections and the spread of antimicrobial resistance but is also a key IPC measure for safe COVID-19 vaccination [4]. ABHR is the preferred method for hand hygiene in health care as it can be easily accessible at the point of care, kills microorganisms quickly (within 20–30 s) and is well tolerated by the skin. These advantages can help to overcome behavioural barriers to compliance. In the light of current shortages, many countries have successfully established local ABHR production as a low-cost alternative within facilities, using WHO-recommended formulations [5].

To highlight the urgent need to save lives by implementing best practices in health care delivery, the slogan for 5 May 2021 is “Seconds save lives—clean your hands” (Fig. 1). The WHO campaign calls to action key stakeholders (Table 1) who can play critical roles in achieving optimal hand hygiene at the point of care, in both the current situation and a broader sense, helping to strengthen society involvement as promoted by the Hand Hygiene for All initiative.

**Fig. 1:**
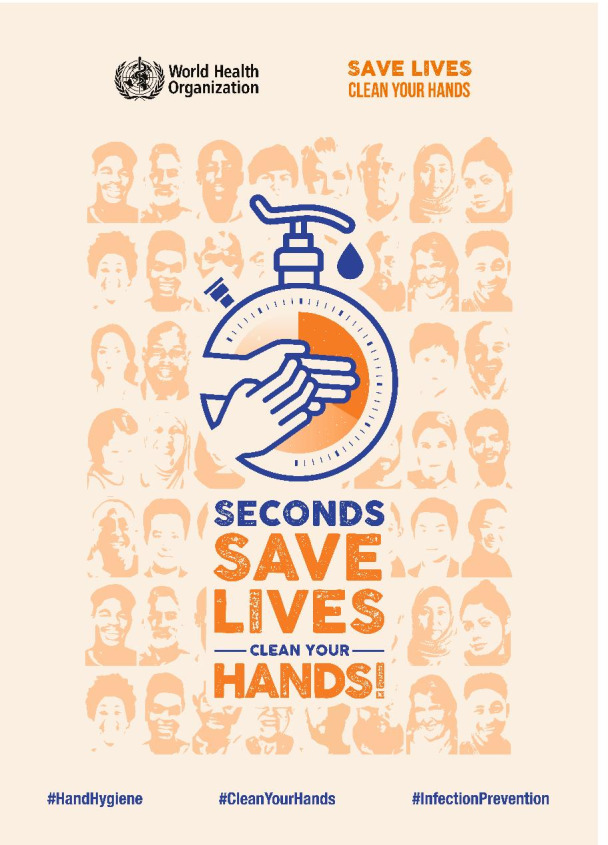
“Seconds save lives—clean your hands”. 5 May 2021 WHO *SAVE LIVES: Clean Your Hands* campaign slogan and main promotional image (2021 hashtags: #HandHygiene, #CleanYourHands, #InfectionPrevention)

**Table 1.** 5 May 2021 *WHO SAVE LIVES: Clean Your Hands* campaign calls to action

Campaign participants	Call to action
Health care workers	Now more than ever, clean your hands at the point of care^a^
IPC^b^ practitioners	Champion and mentor clean hands at the point of care
Facility managers	Ensure hand hygiene supplies are available at every point of care
Policy-makers	Invest now to ensure hand hygiene for all
Patients and families	Help us to help you: please clean your hands
Vaccinators	Clean your hands with every vaccine
General public	Make clean hands your habit—it protects us all

^a^Point of care refers to the to the place where three elements come together: the patient, the health care worker, and care or treatment involving contact with the patient or their surroundings (as published in the WHO guidelines on hand hygiene in health care)

^b^*IPC* infection prevention and control

## Data Availability

Not applicable.

## Abbreviations

WHO: World Health Organization
WASH: Water, Sanitation and Hygiene
ABHR: Alcohol-based handrub
IPC: Infection prevention and control
